# Delegates to the Convention for Revising the National Pharmacopœia

**Published:** 1850-03

**Authors:** George B. Wood

**Affiliations:** Vice President of the Pharm. Convention of 1840; Philadelphia


DELEGATES TO THE CONVENTION FOR REVISING THE NATIONAL PHARMACOPŒIA.

In compliance with a resolution of the Convention for revising the National Pharmacopœia, which met at Washington on the 1st of January, 1840, the Vice President announces the appointment of the following delegates to the ensuing Convention, to be held at Washington, *on the first Monday in May*, 1850. The names are given in the order in which they were received by him.

From "the Faculty of the National Medical College," at Washington—Professors Riley, Miller, and Foreman.

From "the Faculty of the Jefferson Medical College" of Philadelphia—Professors Franklin Bache, and R. M. Huston.

From "the College of Pharmacy of the City of New York,"—Messrs. John Milhau, George D. Coggeshall, and James S. Aspinwall.

From the Medical Faculty of "the University of Pennsylvania,"—DRS. GEORGE B. WOOD, and JAMES B. ROGERS.

From the "College of Physicians of Philadelphia,"—DRS. JOSEPH CARSON, HENRY BOND, and FRANCIS WEST.

From "the Geneva Medical College,"—PROFESSOR JAMES M. BRYAN, M.D.

From "the New Hampshire Medical Institution,"—DRS. ALBERT SMITH, and DIXI CROSBY.

From "the Philadelphia College of Pharmacy,"—WM. PROCTER, JR., DANIEL B. SMITH, and CHARLES ELLIS.

From "the Rhode Island Medical Society,"—DRS. USHER PARSONS, and DAVID KING.

From "the New Jersey State Medical Society,"—DRS. L. CONDICT, and W. A. NEWELL.

From "the Faculty of the Medical Department of the University of Buffalo,"—DR. CHARLES A. LEE.

GEORGE B. WOOD,
Vice President of the Pharm. Convention of 1840.

*Philadelphia*, Feb. 25th, 1850.

